# Review of mechanisms and frontier applications in IL-17A-induced hypertension

**DOI:** 10.1515/med-2025-1159

**Published:** 2025-02-27

**Authors:** Ruiyuan Li, Lipeng Guo, Bin Liang, Wei Sun, Feng Hai

**Affiliations:** Graduate School of Jinzhou Medical University, Jinzhou, Liaoning, China; Department of Cardiology, Dalian Third People’s Hospital of Jinzhou Medical University, Dalian, 116033, Liaoning, China; Department of Critical Care Medicine, Dalian Third People’s Hospital of Jinzhou Medical University, Liaoning, China; Department of Cardiology, Dalian Third People’s Hospital of Jinzhou Medical University, No. 40 Qianshan Road, Dalian, 116033, Liaoning, China

**Keywords:** hypertension, interleukin 17A, immunity, T helper 17 cells, inflammation

## Abstract

**Background:**

The immune system is closely related to hypertension. Hypertension is an immune disorder to a certain extent, and inflammation is the basis of abnormally elevated blood pressure (BP). The accumulation of T cells and their cytokines can increase BP and end organ damage. T cells are activated by antigen-presenting cells of the innate immune system or by the influence of a high-sodium diet, the self-environment, or the gut microbiota. These cells produce inflammatory factors and cytokines, such as interleukin-17A (IL-17A) in T helper 17 cells, causing vascular inflammation, hypertension, and target organ damage

**Methods:**

In this article, we provide an insightful review of the research progress regarding the role of IL-17A in the pathogenesis of hypertension and its effects on different organs while emphasizing the role of IL-17A and its mediated functions in the kidneys, brain, intestines, and vascular system in the development and progression of hypertension.

**Results:**

At the organ level, IL-17A is involved in the development and progression of hypertension in the kidneys, brain, intestines, and blood vessels, interacting with multiple signal pathway.

**Conclusions:**

These findings have significant implications for developing future immunomodulatory therapies, which may lead to the development of potential treatments for hypertension.

## 1 Introduction

Recent studies have demonstrated the involvement of interleukin-17A (IL-17A) in the progression of autoimmune diseases such as ankylosing spondylitis [1] and psoriatic arthritis (PsA) [2] with anti-IL-17A treatment found to mitigate autoimmune disease progression [3] Moreover, IL-17A is also closely associated with cardiovascular diseases such as atherosclerosis [4,5] and heart failure [6,7], and prostate cancer [8,9]. Thus, elevated IL-17A levels may serve as a marker for cardiovascular event risk assessment. There is limited information on the mechanism underlying the association between IL-17A and hypertension, and there are no comprehensive reviews of the effects of IL-17A on different systems in the pressor response. The present article systematically analyzes the relationship between IL-17A and hypertension in terms of the source, production, and actions of IL-17A, together with an analysis of the underlying mechanism, current research status, and cutting-edge applications of IL-17A in the pressor response in multiple systems.

## 2 Source and regulatory pathways of IL-17A

### 2.1 General biology of IL-17A

In 2005, the T helper 17 (Th17) cell emerged as a new T cell subset that can produce a unique proinflammatory cytokine, interleukin-17 (IL-17) [10]. Unlike other IL-17 isoforms, IL-17A is currently considered the member of the IL-17 family that is most involved in autoimmune diseases and the most deeply studied member in various diseases. In the state of autoimmune activation or excessive dietary sodium, peroxidation caused by aseptic inflammatory injury causes professional antigen-presenting cells (APCs), including dendritic cells, macrophages, and monocytes, to secrete prohypertensive cytokines, such as IL-6, IL-1β, and tumor necrosis factor alpha (TNF-α). APCs process exogenous proteins and modified self-proteins into peptides, leading to the formation of modified proteins, such as IsoLG protein adducts, and interactions between CD28/B7 ligands and CD27/CD70 [11]. This process stimulates the proliferation of T cells and the polarization of Th17 cells to produce IL-23 and interferon gamma.

The differentiation of mature Th17 cells from naive Th17 cells is normally controlled by master transcriptional regulators – retinoic acid receptor-related orphan nuclear receptor gamma (RORγt) and signal transducer and activator of transcription 3 (STAT3) [12]. Th17 cell differentiation is induced by transforming growth factor beta (TGF-β) and IL-6. TGF-β promotes RORγt expression of Th17 cells, while IL-6 and IL-1β amplify the Th17 lineage. IL-23 induces RORγt maturation and expansion. IL-21 is produced by Th17 cells and drives IL-17A production in a STAT3-dependent manner. The presence of IL-1β and IL-23 maximizes the expression of IL-17A [13]. IL-17A regulates downstream cells by interacting with the IL-17A receptor. Upon binding of IL-17A to its receptor, signal proteins downstream of the IL-17A receptor are activated, leading to the activation of transcription factors, such as nuclear factor kappa B (NF-κB), activator protein 1 (AP-1), and CCAAT/enhancer-binding protein.

### 2.2 Regulation of IL-17A in hypertensive states

Chronic elevated circulating levels of IL-17A could contribute to organ damage and hypertension. Intravenous injection of IL-17A was found to increase blood pressure (BP) and heart rate, and similar results occurred after paraventricular nucleus (PVN) microinjection or intracerebroventricular (ICV) [14]. The results of kidney biopsies of hypertensive nephrosclerosis patients showed that Th17 and γδT lymphocytes existed in kidney tissue, which were IL-17A-positive cells [15].

IL-17A promotes the elevation of BP through three intracellular signaling pathways: Janus kinase 1 [16], Janus kinase 2 [17], and phosphoinositide 3-kinase (PI3K) [18]. The major T cell sources of IL-17A in hypertensive target organs are CD4^+^ Th17 cells and γδT cells. It should be noted that IL-17A can also be produced by other cells under certain conditions, including natural killer cells, lymphoid tissue inducer cells, and group 3 innate lymphoid cells [19], but the relative contribution of these cells to total IL-17A production in the development of hypertension is unknown. IL-17A can activate and recruit neutrophils in blood vessels and central glial cells, upregulate the expression of inflammatory mediators, promote inflammation, damage the vascular endothelium, and cause vascular dysfunction. IL-17A induces oxidative stress injury and endothelial dysfunction, which contributes to hypertension.

In addition, IL-17A is also reported to affect other cardiovascular risk factors. Increased serum IL-17A is regarded as a risk factor for autoimmune type 1 diabetes for the Chinese population [20]. Depletion of IL-17A ameliorated retinal inflammation, oxidative stress, and vascular leakage in diabetic retinopathy [21]. Il-17A is also reported to be an independent risk factor for dyslipidemia among AR patients. Apparently, IL-17A is an important factor that not only regulates hypertension but also affects other disease factors that may cause hypertension.

## 3 Mechanisms of IL-17A-induced hypertension through multiple pathways

### 3.1 Renal mechanisms

The kidney is the most commonly affected end-target organ of hypertension and is closely related to the development and progression of hypertension. The kidney is not only an organ for excreting metabolites, but also an endocrine organ that regulates water and electrolyte balance, regulates BP, and maintains homeostasis in the internal environment. Therefore, the kidneys play an important role in the regulation of BP.

#### 3.1.1 By regulating sodium/hydrogen exchanger isoform 3 (NHE3), sodium/chloride cotransporter (NCC), and Na^+^−K^+^−2Cl^−^ cotransporter 1 (NKCC1) activity

IL-17A produced by immune cells was found to induce serum- and glucocorticoid-inducible kinase 1 (SGK1)-dependent expression and activity of proximal and distal sodium transporters (including NHE3 and NCC) in the kidneys, resulting in increased sodium and water retention. In addition, IL-17A also impairs glomerular selectivity and tubular secretory activity, thereby contributing to increased BP and impaired renal function. At present, IL-17A is the only IL-17 family member that has a unique regulatory effect on NHE3 and NCC in the proximal and distal tubules [22]. This may be related to the activation of the Rac1-mineralocorticoid receptor (MR)-SGK1-NCC and beta-2 adrenergic and glucocorticoid receptor-with-no-lysine kinase 4-NCC pathways after excessive activation of the renal sympathetic nervous system following upregulation of salt loading [23,24]. Rac1 is a member of Ras-related C3 botulinum toxin substrate (Rac) GTPases, acting as a molecular switch regulating different cellular functions. It is associated with reactive oxygen species (ROS) production, apoptosis, etc. [25]. The MR is a ligand-activated transcription factor, which is reported to be activated by Rac1 [26]. Researchers found that constitutively active Rac1 promoted gene transcription dependent on the MR and the nuclear translocation of the MR, finally aggravating podocyte injury. The serum/glucocorticoid regulated kinase 1 (SGK1) is a significant molecule regulating signal transduction pathways and cell phosphorylation cascades [20]. It is also reported that SGK1 was able to phosphorylate NEDD4 and induce inflammatory fibrosis and hinder Treg development [27]. In the salt-sensitive hypertension animal model, Rac-1 was also found to amplify MR activation and promote its nuclear translocation [28]. The abnormal activation of the Rac-1-MR pathway led to sodium reabsorption via NCC in distal convoluted tubule 2 segment [29]. Correspondingly, when the uptake of sodium increases, SGK is also upregulated by MR [30] Under salt-sensitive conditions, sympathetic nervous system overactivation increases NCC activity, renal sodium reabsorption, and BP by upregulating SGK1, a renal NCC and epithelial sodium channel (ENaC) activator, and downregulating WNK4, whose activation leads to renal sodium excretion. Meanwhile, Norlander et al. [22] found that in addition to the production of IL-17A by T cell stimulation, human renal cortical proximal tubular epithelial cells and mouse distal convoluted tubule 15 (mDCT15) cells in the kidney also produce IL-17A.

IL-17A increases NCC activity in mDCT15 cells through the SGK1/Nedd4-2-dependent pathway. Meanwhile, it increases sodium/hydrogen exchange protein activity by increasing SGK1 expression and phosphorylation in the proximal tubule. It is involved in angiotensin II-induced hypertension and kidney damage. In addition, loss of SGK1 can slow down hypertension, eliminate renal and vascular inflammation, protect the hypertensive kidney, and mitigate vascular damage. However, NKCC1 was found to be upregulated in Th17 cells in salt-induced hypertensive mice, and it mediated the salt-induced increase in SGK1 and IL-23 receptors [31].

#### 3.1.2 By inducing renal fibrosis and elevated BP

Renal inflammation, subsequent fibrosis, and impaired function lead to the development of hypertension [32]. Orejudo et al. [33] found that IL-17A is involved in inflammatory cell accumulation in the kidneys and that injection of anti-IL-17A antibodies can reduce the nephritic state of pre-clinical renal injury. First, inhibition of IL-17A reduced renal inflammation. Saleh et al. [34] found that staining for the cell surface markers CD45, CD3, CD4, CD8, and F4/80 on leukocytes in the kidneys of Ang II-induced hypertensive mice that had been treated with monoclonal antibodies to IL-17A and IL-17 receptor A subunit revealed significantly decreased levels of total T cells, CD4^+^ T cells, and CD8^+^ T cells. On the other hand, the accumulation of total leukocytes, T cells, and both CD4^+^ and CD8^+^ T cells in the kidney was decreased after angiotensin II-induced hypertension was blunted, which was induced by phenol application in bilateral renal denervation [35]. Collectively, those data revealed IL-17A might promote renal hypertension via inducing immune cells accumulation like CD4^+^ T cells and CD8^+^ T cells.

**Figure 1:**
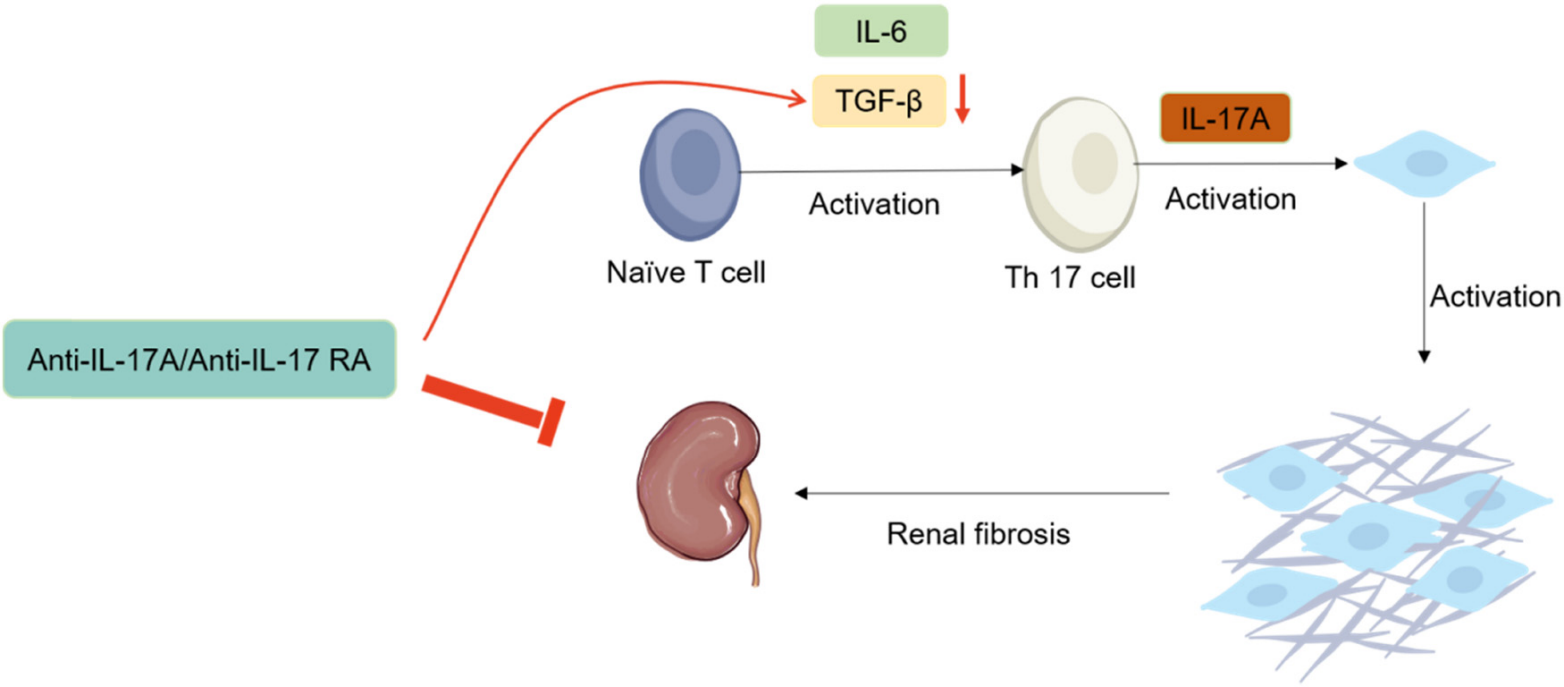
Interaction between IL-17A and other cytokines in renal fibrosis. Naive T-cells develop into Th17 cells under the stimulation of TGF-β and IL-6 and secrete IL-17A. On the other hand, anti-IL-17A or anti-IL-17 receptor A subunit monoclonal antibodies significantly reduced TGF-β levels in renal fibrosis.

In addition, IL-17A regulates renal fibrosis (Figure 1), further influencing BP. Anti-IL-17A or anti-IL-17 receptor A subunit monoclonal antibodies significantly reduced TGF-β levels in TGF-β-mediated renal fibrosis. Naive T-cells develop into Th17 cells under the stimulation of TGF-β and IL-6 and secrete IL-17A [36], which can create a vicious cycle leading to increased fibrosis. More than this, renal fibrosis is caused by the aggregation and activation of fibroblasts regulated by IL-17A, as well as a significant increase in the production of fibrocyte-related chemokine CXCL12 and activating factors Semaphorin7A and PDGF-BB, which significantly induce and participate in the pathogenesis of renal fibrosis [37].

However, there are different perspectives on the role of IL-17A in renal injury. It was found that IL17^−/−^ deficient mice exerted less vascular dysfunction as per Madhur et al. [38]. In this study, the mouse hypertension model was induced only by Angiotensin II. And at the first 2 weeks, there was no significant change in BP between wild-type mice and IL17^−/−^ mice. And by 3–4 weeks of angiotensin II infusion, the BP of IL17^−/−^ mice began to obviously decline. Likewise, the application of IL-17A neutralizing antibody did not show obvious effect on BP or albuminuria [39]. In contrast, Krebs et al. [40] discovered that there was no apparent change in hypertensive response between IL-17^–/–^ and wild-type mice, which was not in line with the previous study. While, more albuminuria and glomerular injury in IL-17^–/–^ mice were found, which was probably induced by γδ T cell infiltration. The increased γδ T cell infiltration was considered as compensatory changes in other immune cell populations. After weeks, the BP was found lower in IL-17^–/–^ mice than in wild-type mice. In this study, the hypertension mouse model was induced by deoxycorticosterone acetate and angiotensin II, which is regarded as inducing substantial hypertensive renal and cardiac injury. Different from the literature mentioned above, the relevant indicators were detected in this study on the 4th and 14th day, which was much earlier than the detection time of the previous study. Compared with those research studies, the difference in model, the frequency and administration of antibodies, and observation period length all contributed to the difference, which needs more samples and long-term observation to confirm.

### 3.2 Central nervous system (CNS)

Numerous studies have shown that inflammation significantly promotes the development of hypertension and heart failure by driving sympathetic hyperactivity and neurohumoral activation; microglia-mediated neuroinflammation plays an important role in this process. In addition, researchers have found that peripheral IL-17A can increase the permeability of the blood–brain barrier (BBB) by reducing the gene expression of tight junction proteins (such as ZO-1, claudin-5, and occludin) and modifying the underlying actin cytoskeleton, which allows IL-17A to enter the brain by damaging the integrity of the BBB [41]. Moreover, studies have also found that IL-17RA and IL-17RC are highly expressed in the CNS, especially in the PVN of the hypothalamus, which lays the foundation for IL-17A to play a role in the brain [14]. Studies have shown that peripheral IL-17A plays a role in promoting chronic inflammation, leading to a pressor response and end-organ damage. There is also strong evidence that brain IL-17A mediates neuroinflammation, and sympathetic outflow also aggravates hypertension and other cardiovascular diseases.

#### 3.2.1 An increase in peripheral IL-17A damages the BBB, activates glial cells in the brain, and promotes an increase in BP

Cao et al. [14] found that in angiotensin II-induced hypertensive rats, intravenous and ICV injections of IL-17A resulted in a significant and sustained increase in BP and excitatory responses, and heart rate and renal sympathetic nerve activity (RSNA) were also maintained at high levels. Systemic administration of IL-17A significantly increased the levels of IL-17A in plasma and cerebrospinal fluid and upregulated the mRNA expression of IL-17A, IL-17F, and IL-17RA in the PVN of the hypothalamus in normal rats. Moreover, the mRNA levels of the inflammatory cytokines TNF-α, IL-1β, IL-6, IL-8 and chemokines CCL2, CCL3, CXCL8, and CXCL12 in the PVN were significantly elevated. In addition, microglia and astrocytes in the PVN showed elevated expression of the surface markers CD11b and glial fibrillary acidic protein. In summary, increased peripheral IL-17A could promote the production of large amounts of IL-17A in the CNS, activate glial cells in the brain, and produce new cytokines or chemokines by these activated glial cells in response to increased IL-17A, which may lead to further BBB damage.

#### 3.2.2 Synergistic effect of IL-17A increases BP and myocardial infarction risk through the CNS

More directly, it was found that injecting trace amounts of IL-17RA small interfering RNA into the PVN significantly reduced BP, heart rate, RSNA, and gene expression of inflammatory cytokines and chemokines. The same effect was also observed in angiotensin II-induced hypertensive rats pretreated with the microglial or astrocyte inhibitors minocycline and fluorouracil [42,43]. IL-17A cooperates with chemokines to amplify its inflammatory effect, and this effect may be the key to promoting sympathetic nerve excitation. The subsequent immune response and tissue inflammation cause a positive feedback loop centered on IL-17A, which aggravates CNS neuroinflammation. ICV administration of IL-17A increased the expression of phosphorylated (p) 44/42 mitogen-activated protein kinase (MAPK), p-TGF-β-activated kinase 1 (TAK1), and phospho-I-kappa B-alpha (IκB-α), and the rapid degradation of IκB-α promoted NF-κB activity [44]. These results suggest that IL-17A activates the TAK1, p44/42 MAPK, and NF-κB signaling pathways in the PVN to promote neuroinflammation, which in turn promotes sympathetic activation and hypertension.

The discovery of the synergistic effects of IL-17A has important implications for cardiovascular diseases, such as hypertension and myocardial infarction. In an experimental study by Yu et al. [45], it was found that in rats with heart failure induced by myocardial infarction, peripheral IL-17A entered the brain and activated autonomic nerve and neuroendocrine neurons in the brain, especially in the PVN, triggering the production of broad spectrum proinflammatory factors and chemokines, thereby activating sympathetic nerve and hormone activation and ultimately leading to the deterioration of cardiac function in heart failure.

#### 3.2.3 Brain–kidney interaction of IL-17A

In line with the above, excessive activation of RSNA promotes the opening of ion channel proteins. Moreover, IL-17A promotes excessive and sustained RSNA by acting on the CNS, which suggests that a brain–kidney interaction may explain the pathogenesis of IL-17A-induced hypertension.

### 3.3 Vascular system

Hypertension is associated with vascular changes that are characterized by endothelial dysfunction, maladaptive vasomotor function, and arterial remodeling. IL-17A plays a key role in the development and progression of hypertension by impairing vascular function through vascular inflammation, promoting an increase in ROS, increasing aortic stiffness, causing vascular fibrosis, and leading to vascular remodeling.

#### 3.3.1 By increasing ROS, vascular fibrosis, arterial stiffness, and arterial remodeling

This ROS-induced mitochondrial ROS release process is directly involved in the prohypertensive response induced by angiotensin II in hypertensive mice [46]. Schuler et al. [47] found an increase in peripheral ROS and significant vascular dysfunction in CD4−IL-17A^ind/+^ mice. Moreover, it was found that overexpression of IL-17A alone led to the formation and increase of peripheral oxidative stress and impaired vascular function. First, IL-17A could disrupt the oxidation-antioxidant balance and downregulate the intravascular soluble guanylate cyclase-cyclic guanosine monophosphate pathway through IL-17A and myeloid cell-mediated oxidative stress, which would impair nitric oxide signaling and increase peripheral vascular resistance. Second, IL-17A is ubiquitously expressed in the vascular wall. It directly induces fibroblast proliferation and vascular collagen deposition through the NF-κB pathway downstream of IL-17A/IL-17RA [48]. Moreover, IL-17A-mediated redox activation activates tyrosine kinase 2 in perivascular adipose tissue, which promotes vascular fibrosis and induces vascular dysfunction. In addition, overexpression of IL-17A also upregulates the profibrotic transcriptional marker matrix metalloproteinase 2 (MMP2) and vascular cell adhesion molecule 1 (VCAM-1) [49]. MMP2 causes fibrosis and hypertrophy of arteries, as well as degradation and expansion of elastic fibers, leading to arterial stiffness and remodeling. VCAM-1 promotes the adhesion and migration of macrophages to the endothelium, leading to the production of various proinflammatory cytokines, including IL-1β, TNF-α, and IL-6, in addition to ROS, which ultimately leads to hypertension [50,51].

#### 3.3.2 By mediating oxidative stress, vascular inflammation, and smooth muscle cell migration and proliferation in a TRAF3IP2-dependent manner

As a potent pro-oxidant and proinflammatory cytokine, IL-17A promotes IL-17A/TRAF3 interacting protein 2 (TRAF3IP2)-mediated oxidative stress, NF-κB, AP-1, and p38MAPK-induced NLRP3 expression, caspase-1 activation, IL-1β and IL-18 secretion, and human primary aortic smooth muscle cell migration and proliferation in a TRAF3IP2-dependent manner [52]. In addition, TRAF3IP2 overexpression impairs insulin signaling in endothelial cells and attenuates endothelial-dependent relaxation in isolated arteries; this inhibition of aortic vasodilation is consistent with the downregulation of endothelial nitric oxide synthase in aortic endothelial cells. However, whether the expression of TRAF31P2 has a significant effect on BP requires more experimental evidence to confirm this finding [53]. Previous studies have shown that IL-17A induces a variety of inflammatory mediators, including IL-6, TNF-α, monocyte chemoattractant protein 1, and TGF-β through TRAF3IP2 to produce and maintain vascular inflammation and damage the vascular endothelium [54].

Clinical data indicated that patients with higher concentrations of IL-17A exerted more severe HF. Subsequent analysis showed elevated activation of lymphocyte-mediated immunity and leukocyte activation pathways in patients with elevated IL-17A [7]. On the other hand, the character of HF is the production and release of proinflammatory cytokines induced by immune activation [55], which will lead to a vicious cycle. In addition, Li et al. discovered that IL-17A damaged cardiac function by cardiac remodeling and calcium handling mediated by NF-κB [6]. More than this, Il-17A also exerted a significant role in ischemic stroke. First, IL-17A accelerated the progress of atherosclerotic plaques and hypertension, which are ischemic stroke risk factors [56]. Second, IL-17A accelerated neuronal injury via mediating neutrophil chemotaxis to the site of injury, the induction of neuronal apoptosis, causing further damage to neurons [57]. Therefore, targeting IL-17A is beneficial not only for the treatment of hypertension, but also for the treatment of complications such as heart failure and stroke.

#### 3.3.3 By regulating Th17/Treg balance

In angiotensin II-infused mice, serum levels of IL-17A, IL-23, and TNF-α, which have roles in maintaining hypertension, were shown to be significantly increased, while serum levels of the anti-inflammatory factor IL-10, which has cardiovascular protective effects, were significantly decreased [58]. Expression of RORγt, a Th17-related transcription factor, was significantly increased, while in contrast, expression of Treg-related transcription factor Foxp3 was significantly decreased. Among these, the SGK1-FoxO1 signaling pathway was shown to be involved in Th17/Treg imbalance and target organ damage in angiotensin II-induced hypertensive mice, and it was also involved in renal/cardiac inflammation and fibrosis induced by vascular endothelial cells in hypertensive mice [59]. Bu-Shen-He-Mai (BSHM) granuleswere found to significantly increase the density of Foxp3, circulating Tregs, and IL-10 in the spleen but decrease the density of RORγt in the spleen, circulating Th17 lymphocytes, IL-6, and IL-17A. BSHM inhibits the inflammatory response and improves the Th17/Treg balance, thereby inhibiting the development and progression of hypertension [60].

### 3.4 Gut microbiota

The gastrointestinal tract is the largest immune organ in the human body, and the gut and gut microbiota are closely related to immune activation and immune regulation. In one study, three-dimensional principal component analysis of fecal bacterial communities in normotensive Wistar Kyoto (WKY) rats and spontaneously hypertensive rats revealed significant differences in microbial community composition between the two groups [61]. Dysbiosis of the gut microbiota contributes to the development of hypertension [62]. Gut microbiota dysbiosis occurs before the development of hypertension, and the gut microbiota has a direct impact on host BP.

#### 3.4.1 By regulating gut microbial immunity

Recent studies have found that as an important part of the gut microbial immune response, IL-17A drives the infiltration and inflammation of vascular immune cells and promotes sodium- and angiotensin II-mediated vascular dysfunction and hypertension [63]. Toral et al. [64] found that transplantation of spontaneously hypertensive rats with fecal microbiota from normotensive WKY rats reduced BP and nicotinamide adenine dinucleotide phosphate (NADPH) oxidase activities, ameliorated IL-17A-mediated immune cell infiltration, and improved angiotensin II-induced endothelial dysfunction and cardiorenal hypertrophy. The Th17/Treg balance in mesenteric lymph nodes and the aorta was restored in angiotensin II-induced hypertensive germ-free mice, which exhibited attenuated IL-17A-induced leukocyte adhesion, reduced vascular and renal inflammatory infiltration, and decreased BP compared with conventional mice [65]. In summary, increased intestinal permeability allows bacteria, as well as IL-17A and other inflammatory factors, to enter the systemic circulation and reach the kidneys, which aggravates renal inflammation and renal damage under hypertension, resulting in impaired glomerular selectivity and tubular secretory activity and an imbalance in internal environmental homeostasis.

#### 3.4.2 By affecting intestinal probiotics

Probiotics and prebiotics can prevent the occurrence of dysbiosis and vascular endothelial dysfunction and alleviate hypertension in hypertensive patients [66]. A high-sodium diet can significantly reduce the survival rate of the Lactobacillus intestinal population and increase intestinal permeability. Supplementation with Lactobacillus as a probiotic can prevent the development of salt-sensitive hypertension by reducing the polarization of Th17 cells and the production of IL-17A through Lactobacillus-induced indole-3-lactic acid [67]. In addition, lactulose can be added to the diet as a prebiotic to maintain a healthy intestinal microenvironment. Compared with untreated high-salt diet mice, high-salt diet mice treated with lactulose showed significant reductions in IL-17A mRNA levels in the small intestine, serum IL-17A and IL-22 levels, inflammatory cytokine levels, and inflammatory cell infiltration. They also showed increased fecal sodium excretion, relieved constipation, regulated intestinal flora, and reduced intestinal permeability. Lactulose also alleviated salt-sensitive hypertension [68].

#### 3.4.3 Pressor role of IL-17A in the interaction between the gut and brain

Recent studies have found that the gut microbiota and CNS regulate BP through two-way communication. Increased neural activity in the CNS promotes enhanced intestinal sympathetic drive, gut dysbiosis, and increased intestinal permeability and inflammatory states. In contrast, fecal transplantation from normotensive WKY rats in the spontaneously hypertensive rat group significantly reduced BP, PVN inflammation, and central sympathetic excitation [37]. IL-17A can directly act on sympathetic nerve axons and nerve endings, promote growth, and increase the permeability of the BBB and the small intestinal epithelial barrier [69]. The immunosuppressive agent mycophenolate mofetil can reduce immune cell infiltration and NADPH oxidase activity in the PVN of the hypothalamus, reduce IL-17A-induced neuroinflammation and sympathetic remodeling during inflammation, improve intestinal integrity, dysbiosis, and aortic endothelial function, and reduce arterial BP in hypertensive rats [70].

**Figure 2:**
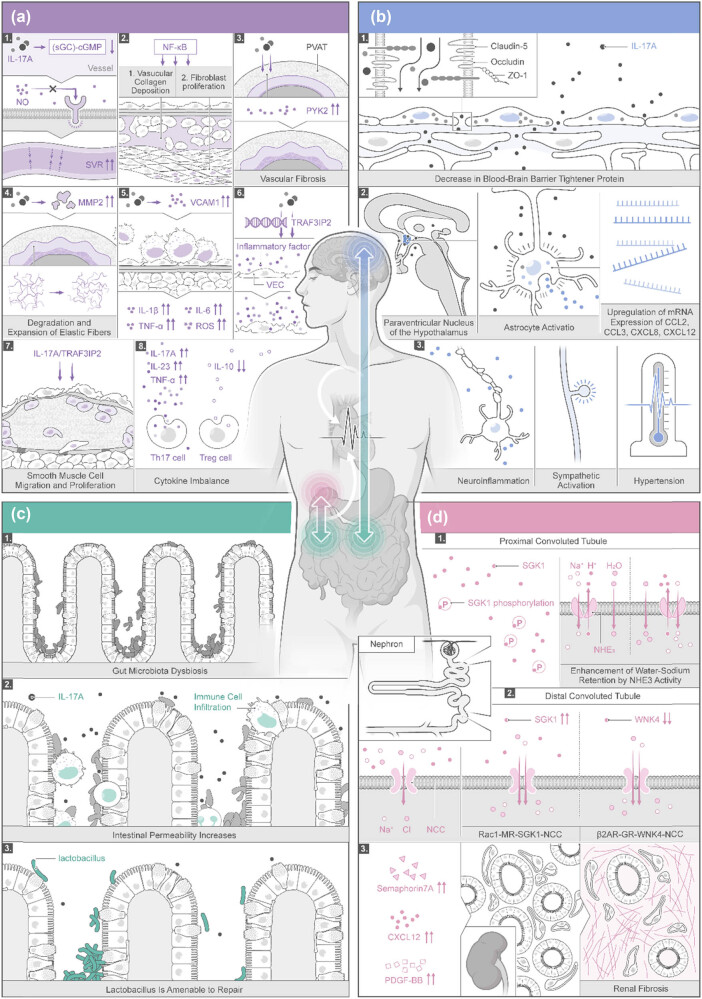
Mechanisms of IL-17A-induced hypertension through multiple pathways in the vascular system (a), brain (b), intestinal microbiota (c), and kidneys (d). When the inflammation from gut microbiota gets further into the circulating bloodstream, the brain, autonomic nervous system, and kidneys will be affected.

Collectively, the gut microbiota activates immune cells, leading to low-grade inflammation that circulates, affecting the brain, autonomic nervous system, and kidneys (Figure 2). The amplified cascade of sympathetic nerve activity and vascular immune inflammation in the nervous system connects the kidneys, CNS, intestines, and blood vessels and then participates in the development and progression of hypertension through target organs, such as the kidneys, brain, intestines, and blood vessels. The role of IL-17A in the treatment of hypertension still needs to be proven by a large number of experiments. The mechanism of hypertension induced by organs such as the kidneys, CNS, intestines, and blood vessels is not a one-to-one connection but a mutual interaction among several organs to promote an increase in BP.

## 4 Anti-IL-17A in the treatment of hypertension

### 4.1 Application of IL-17A inhibitors

As a potential biological marker of cardiovascular risk, IL-17A is closely related to cardiovascular diseases, such as atherosclerosis, heart failure, viral myocarditis, and hypertension. Anti-IL-17A therapy can not only improve the skin manifestations of psoriasis but also improve cardiovascular inflammation, vascular function, and metabolic factors in the treatment of autoimmune diseases, such as ankylosing spondylitis, and PsA [71]. From the perspective of clinical trials of anti-cytokine drugs, it has been found that canakinumab, a monoclonal antibody targeting IL-1β, successfully prevented cardiovascular events in patients with a history of myocardial infarction [72]. IL-1 receptor antagonists have been reported to reduce BP in obese individuals [73]. Specific monoclonal antibodies directly targeting IL-17A (secukinumab and ixekizumab) and antibodies and drugs acting on IL-17A and IL-17F (bimekizumab) have been used in the clinical treatment of psoriasis with hypertension and other cardiovascular diseases, as well as other cardiovascular risk factors. They have achieved significant benefits for patients with cardiovascular diseases [74,75]. In addition, statins can reduce the proinflammatory and prothrombotic effects of IL-17A and TNF-α on endothelial cells by acting through the cholesterol pathway [76]. Metformin treatment reduced IL-17A expression, mediated redox balance, add an anti-inflammatory effect, and protected endothelial function [77,78].

In the mouse neurovascular coupling (NVC) injured model induced by angiotensin (Ang) II in the context of hypertension, IL-17A promoted the generation of cerebral superoxide anion production. While the application of neutralization of IL-17A or specific inhibition of its receptor ameliorated NVC damage [79]. However, Jiang et al. found anti-IL-17 treatment might escalate hypertension risk [80]. They conducted a meta-analysis on 9,909 patients with diverse autoimmune diseases who received anti-IL-17 agents, which revealed only secukinumab exhibited a notable association with hypertension among secukinumab, ixekizumab, bimekizumab, and brodalumab these four agents.

### 4.2 Safety of IL-17A inhibitors

It is well recognized that IL-17 pathway plays a significant role in defending against non-system fungal infections [81]. IL-17A inhibition usually leads to adverse events like paradoxical psoriasis [82] and atopic-like eczema [83]. At present, most of the side effects of treatment are found in the treatment of other diseases, such as ankylosing spondylitis [84], PsA [85], etc. The safety of IL-17A inhibitors needs a larger sample size and longer follow-up times to be verified. As IL-17A is rarely used in hypertensive diseases, the side effects of hypertension remain to be further explored.

In summary, anti-IL-17A therapy may provide a new perspective for the prevention of inflammatory diseases and the treatment of hypertension in the general population. Improvements in cardiovascular events may be a direct result of specific or relatively nonspecific anti-inflammatory treatments. Whereas, current research about the application of IL-17A inhibitors in hypertension is relatively rare. Whether in basic research or clinical trials, IL-17A inhibitors have not been used in hypertensive diseases. From the current research results, first, the effective and safe dose of IL-17A inhibitor is uncertain, and second, the application of the inhibitor in multiple diseases cannot be determined that it plays an effective protective role in multiple diseases.

## 5 Conclusions

In this review, the various ways in which IL-17A regulates BP are summarized systematically at molecule level, such as sodium and water retention, central sympathetic outflow and excitability, oxidative stress, vascular fibrosis, arterial stiffness, vascular dysfunction, and intestinal flora imbalance. At the organ level, IL-17A participates in and mediates the development and progression of hypertension in the kidneys, brain, intestines, and blood vessels. The current study suggests that IL-17A may play an important role in salt-sensitive hypertension, and remains to be studied in other types of hypertension. Novel therapies targeting IL-17A signaling have been approved for the treatment of autoimmune diseases and have shown promise in both animal models and human studies of hypertension. However, further investigation is required into these drugs to determine their specific suitability for the treatment of high BP. At present, there are clear clinical applications of successful IL-17A target therapy standards for the treatment of autoimmune diseases. However, no clinical studies have reported the therapeutic effect of targeting IL-17A in hypertensive patients, so relevant clinical cohort studies are needed to fill this gap.

Since IL-17A plays an effective role in the treatment of autoimmune diseases, hypertensive diseases, and tumor suppression, targeting it may have multiple protective effects in clinical therapy. It will also be an important research direction to apply it to other IL-17A-based cell-targeted biotherapy treatments currently under development and to target more specifically on IL-17A for the treatment of hypertension.
